# Expression, regulation and activity of a B2-type cyclin in mitotic and endoreduplicating maize endosperm

**DOI:** 10.3389/fpls.2014.00561

**Published:** 2014-10-17

**Authors:** Paolo A. Sabelli, Ricardo A. Dante, Hong N. Nguyen, William J. Gordon-Kamm, Brian A. Larkins

**Affiliations:** ^1^School of Plant Sciences, University of ArizonaTucson, AZ, USA; ^2^Pioneer Hi-Bred International Inc., Johnston, IOUSA

**Keywords:** cell cycle, cyclin, cyclin-dependent kinase, endoreduplication, endosperm, maize, proteasome

## Abstract

Cyclin-dependent kinases, the master regulators of the eukaryotic cell cycle, are complexes comprised of a catalytic serine/threonine protein kinase and an essential regulatory cyclin. The maize genome encodes over 50 cyclins grouped in different types, but they have been little investigated. We characterized a type B2 cyclin (CYCB2;2) during maize endosperm development, which comprises a cell proliferation phase based on the standard mitotic cell cycle, followed by an endoreduplication phase in which DNA replication is reiterated in the absence of mitosis or cytokinesis. *CYCB2;2* RNA was present throughout the period of endosperm development studied, but its level declined as the endosperm transitioned from a mitotic to an endoreduplication cell cycle. However, the level of CYCB2;2 protein remained relatively constant during both stages of endosperm development. CYCB2;2 was recalcitrant to degradation by the 26S proteasome in endoreduplicating endosperm extracts, which could explain its sustained accumulation during endosperm development. In addition, although CYCB2;2 was generally localized to the nucleus of endosperm cells, a lower molecular weight form of the protein accumulated specifically in the cytosol of endoreduplicating endosperm cells. In dividing cells, CYCB2;2 appeared to be localized to the phragmoplast and may be involved in cytokinesis and cell wall formation. Kinase activity was associated with CYCB2;2 in mitotic endosperm, but was absent or greatly reduced in immature ear and endoreduplicating endosperm. CYCB2;2-associated kinase phosphorylated maize E2F1 and the “pocket” domains of RBR1 and RBR3. CYCB2;2 interacted with both maize CDKA;1 and CDKA;3 in insect cells. These results suggest CYCB2;2 functions primarily during the mitotic cell cycle, and they are discussed in the context of the roles of cyclins, CDKs and proteasome activity in the regulation of the cell cycle during endosperm development.

## INTRODUCTION

The eukaryotic cell division cycle is driven by the activity of complexes between a serine/threonine cyclin-dependent kinase (CDK) and a regulatory cyclin (CYC) protein. Periodic activation of different CDK/CYC complexes ensures the typically unidirectional execution of cell cycle steps, such as DNA replication and chromosome segregation (mitosis), and progress through their intervening transitions. A number of mechanisms are known to regulate CDK/CYC activity, including gene transcription, phosphorylation, and dephosphorylation of key amino acids, proteolysis by the 26S proteasome, and binding by specific inhibitors (De Veylder et al., 2007; Harashima et al., 2013). Compared to yeast and mammals, plant genomes encode a larger number of these two components of CDK complexes, and it has been challenging to dissect their function, especially because of the genetic redundancy within the two protein families. Understanding the mechanisms governing the cell cycle, coupled with the ability to manipulate it, offer the potential to enhance crop performance and yield, and several important examples are known in which alteration of cell cycle control has significantly impacted crop evolution and breeding (Sabelli, 2014). Although the cell cycle is relatively well characterized in the model species *Arabidopsis thaliana*, our understanding of cell cycle control in economically important crops, and particularly in major grasses such as maize, rice and wheat, is still in its infancy, and there is a pressing need to unravel the roles of key cell cycle-controlling genes in these crops. Because yield in maize and related cereals is in part determined by grain size and weight, it is important to understand how different cell cycle genes are regulated and how they function during seed development.

The most economically important seed tissue is the endosperm, which originates at fertilization by the fusion of one sperm cell nucleus with two polar nuclei within the central cell of the female gametophyte (reviewed in Becraft et al., 2001; Olsen, 2004; Sabelli and Larkins, 2009c). In maize, the primary triploid endosperm nucleus goes on to proliferate by acytokinetic mitoses to generate a syncytium. Deposition of cell wall material between nuclear domains followed by cell proliferation through mitotic cell division give rise to virtually all the endosperm cells by approximately 12 days after pollination (DAP). However, starting from around 8–10 DAP, inner cells of the endosperm switch to an endoreduplication type of cell cycle, which is characterized by reiterated rounds of DNA synthesis in the absence of mitosis or cell division; typically, this results in large, polyploid nuclei and cells. Endoreduplication proceeds toward the periphery of the endosperm and is roughly coincident with the massive accumulation of storage compounds, such as starch and zein proteins. From around 16 DAP, central endosperm cells begin to undergo programmed cell death, and this continues so that at seed maturity all endosperm cells are dead, except for the peripheral aleurone layer. Because the mitotic phase of endosperm development produces virtually all the cells involved in storage compound accumulation and because the endoreduplication phase is associated with rapid cell growth and endosperm expansion and filling, it is important to understand how the cell cycle is regulated during these two phases (Sabelli and Larkins, 2009b). In this context, three maize CDKs: CDKA;1, CDKA;3, and CDKB1;1; several cyclins belonging to the A1, B1, D2, and D5 types (Grafi and Larkins, 1995; Sun et al., 1999b; Leiva-Neto et al., 2004; Dante et al., 2014); two CDK inhibitors (Coelho et al., 2005); a Wee1 homolog (Sun et al., 1999a); and members of the retinoblastoma-related (RBR) protein family (Grafi et al., 1996; Sabelli et al., 2005, 2013) have been studied in some detail.

Recent genome-wide analyses revealed the cyclin family in maize comprises over 50 members (Hu et al., 2010), and for the most part these genes have not been investigated. B-type cyclins are believed to be primarily involved in the regulation of M-phase and appear to play important roles in plant growth and seed development. CYCBs are known to stimulate cell division and tissue growth in ectopic or over-expression studies (Doerner et al., 1996; Lee et al., 2003). Knockdown of CYCB1;1 in rice caused aborted seed endosperm due to abnormal cellularization of the syncytium (Guo et al., 2010) and resulted in large, triploid embryo cells (Guo et al., 2014). In addition, CYCB2;2 was implicated in the timing of rice endosperm cellularization, cell number, and grain size and yield (Ishimaru et al., 2013). Here we describe CYCB2;2 from maize with respect to its spatiotemporal expression patterns, interaction with CDKs, associated kinase activity, and proteolysis patterns during endosperm development. These data suggest this cyclin likely functions during cell division and cell wall formation in mitotic cells and becomes stabilized in endoreduplicating cells with the accumulation of a lower molecular weight form in the cytoplasm.

## MATERIALS AND METHODS

### PLANT MATERIALS

Maize (*Zea mays* L.) B73 plants were grown in the field or a greenhouse and hand-pollinated. Endosperms were dissected from kernels harvested at different stages of development and processed for molecular and immunohistochemical analyses as described in previous publications (Leiva-Neto et al., 2004; Sabelli et al., 2005, 2013; Dante et al., 2014).

### DATABASE SEARCHES AND SEQUENCE ANALYSES

A nucleotide sequence encoding CYCB2;2 was initially obtained by querying Pioneer Hi-Bred’s maize EST database. Additional searches were made in the Maize Genome Sequencing Project^1^, MaizeGDB^2^ (Lawrence et al., 2004), Phytozome v10^3^, Rice Genome Annotation Project v7^4^ (Kawahara et al., 2013), Gramene v42^5^ (Ware et al., 2002), and Pfam v27^6^ (Finn et al., 2014) databases and the Maize eFP Browser^7^ (Sekhon et al., 2011). Functional motifs were predicted with the Eukaryotic Linear Motif Resource^8^ (Dinkel et al., 2014). Subcellular localization was predicted using LocTree 3^9^ (Goldberg et al., 2012), Plant-mPLoc^10^ (Chou and Shen, 2010), and BaCelLo^11^ software. Multiple sequence alignments were carried out with M-Coffee^12^ (Di Tommaso et al., 2011) or MUSCLE (Edgar, 2004). An un-rooted Neighbor-Joining tree of a set of 35 plant B-type cyclin amino acid sequences, spanning the conserved Cyc_N and Cyc_C domains (Nugent et al., 1991), was constructed using MEGA6 software package (Tamura et al., 2013). Amino acid sequences were selected based on previous analyses (La et al., 2006; Guo et al., 2007; Hu et al., 2010; Jia et al., 2014) and novel database searches. Only one amino acid sequence per locus was selected in the case of multiple predicted transcripts. Several shorter amino acid sequences (GRMZM2G025200_P01, Loc_Os02g41720, AT1G34460, Sb07g003015, Potri.006G035200.2) were not included in the analysis. The evolutionary distances were computed using the Poisson correction method. All positions containing gaps and missing data were eliminated. There were a total of 235 positions in the final dataset.

### ANALYSES OF ENDOSPERM RNA AND PROTEINS

Detailed procedures for purification of endosperm RNA and protein and their analyses by RT-PCR and immunoblotting, respectively, are given in previous publications (Sabelli et al., 2005, 2013; Dante et al., 2014). The following RT-PCR primers were used for CYCB2;2: CYCB2;2F (GAAAATGAGGCTAAGAGTTGTGTAAG) and CYCB2;2R (GAGCTCCAGCATGAAAAATGACGCT) and actin: ACT1-F (ATTCAGGTGATGGTGTGAGCCACAC) and ACT1-R (GCCACCGATCCAGACACTGTACTTCC). Each developmental stage comprised a pool of 5–13 endosperm RNA samples. Two analysis replicates were carried out and the RNA levels averaged, normalized to those of actin control, and displayed relative to those at 7-DAP.

Analysis of *CYCB2;2* RNA accumulation patterns in 14 different tissues/developmental stages was carried out by compiling Nimblegen-derived RNA expression data from Sekhon et al. (2011), available at the Maize eFP Browser^7^.

Immunohistochemical localization assays were carried out essentially as described by Dante et al. (2014), except for a monoclonal anti-tubulin antibody (YOL 1/34, Accurate Chemical and Scientific Corp., Westbury, NY, USA), which was used to stain microtubules. Antibodies were utilized at a concentration of 0.5–1 μg/ml. Immunoprecipitation and kinase activity assays were carried out as described previously (Dante et al., 2014).

### PROTEIN EXPRESSION IN HETEROLOGOUS SYSTEMS AND ANTIBODIES

Sequences encoding selected domains of maize cyclins (amino acid residues 1–243 of CYCA1;1, 1–206 of CYCB1;3, and 4–143 of CYCB2;2) were expressed as GST fusions in *Escherichia coli* and purified according to procedures described previously (Dante et al., 2014). For CYCB2;2, a cDNA fragment encoding a 139-aa long, *N*-terminal domain was amplified with primers B2;2 4–143F (ATCGCGGATCCCGCGCGGCGGATGAAAACCGCAGACC) and B2;2 1–62R (CCGCTCGAGTCAGAGCAATTCATCTTCGTTCATAATGTCC), and cloned at the *Bam*HI and *Xho*I sites of the pGEX4T-3 vector (GE Heathcare, Life Sciences). Antibodies were generated in rabbits (anti-cyclin) and affinity purified or purchased (anti-actin) as previously reported (Dante et al., 2014).

For expression in *Drosophila* S2 cells, CYCB2;2 was PCR-amplified with primers E2-KpnI (ATCCGCGGTACCAAACATGGCGGCGCGGGCGGCTGACGAGAAC) and E2HAS2 (ATCGC GAATTCCTATTAGGCGTAATCGGGCACATCGTAGGGGTAG-TTTGCACCTGAAGGAGGCGG), cloned into the *Kpn*I and *Eco*RI sites of the pHSKSMCS vector (kindly provided by Dr. Thomas Bunch, University of Arizona), expressed in *Drosophila* S2 cells either alone or co-expressed with maize CDKA;1 or CDKA;3, and analyzed essentially as described previously for other cyclins (Dante et al., 2014). CYCB2;2 was immunoprecipitated from cell lysates and assayed for interaction with co-expressed maize CDKs using an anti-PSTAIR (Sigma, catalog No. P7962) antibody (Dante et al., 2014). Immunoprecipitates were also tested for kinase activity on histone H1 substrate in *in vitro*-assays as described (Dante et al., 2014).

### PROTEIN STABILITY ASSAYS

^35^S-radiolabeled CYCB2;2, and a T417A mutant (CYCB2;2T/A) polypeptides were synthesized *in vitro* using TNT T7 Quick for PCR DNA kit (Promega, Madison, WI, USA) as described (Dante et al., 2014). These proteins were incubated with cell extracts obtained from prevalently mitotic (i.e., 7-DAP) or endoreduplicating (i.e., 15-DAP) endosperm for 90 min before being subjected to SDS-PAGE and authoradiography. The 26S proteasome-specific inhibitor, carboben-zoxyl-leucinyl-leucinyl-leucinal (MG-132, Sigma), was utilized to determine whether protein degradation was proteasome-dependent. Procedures for these assays were as previously reported (Dante et al., 2014).

## RESULTS

### IDENTIFICATION OF CYCB2;2 FROM MAIZE

**FIGURE 1:**
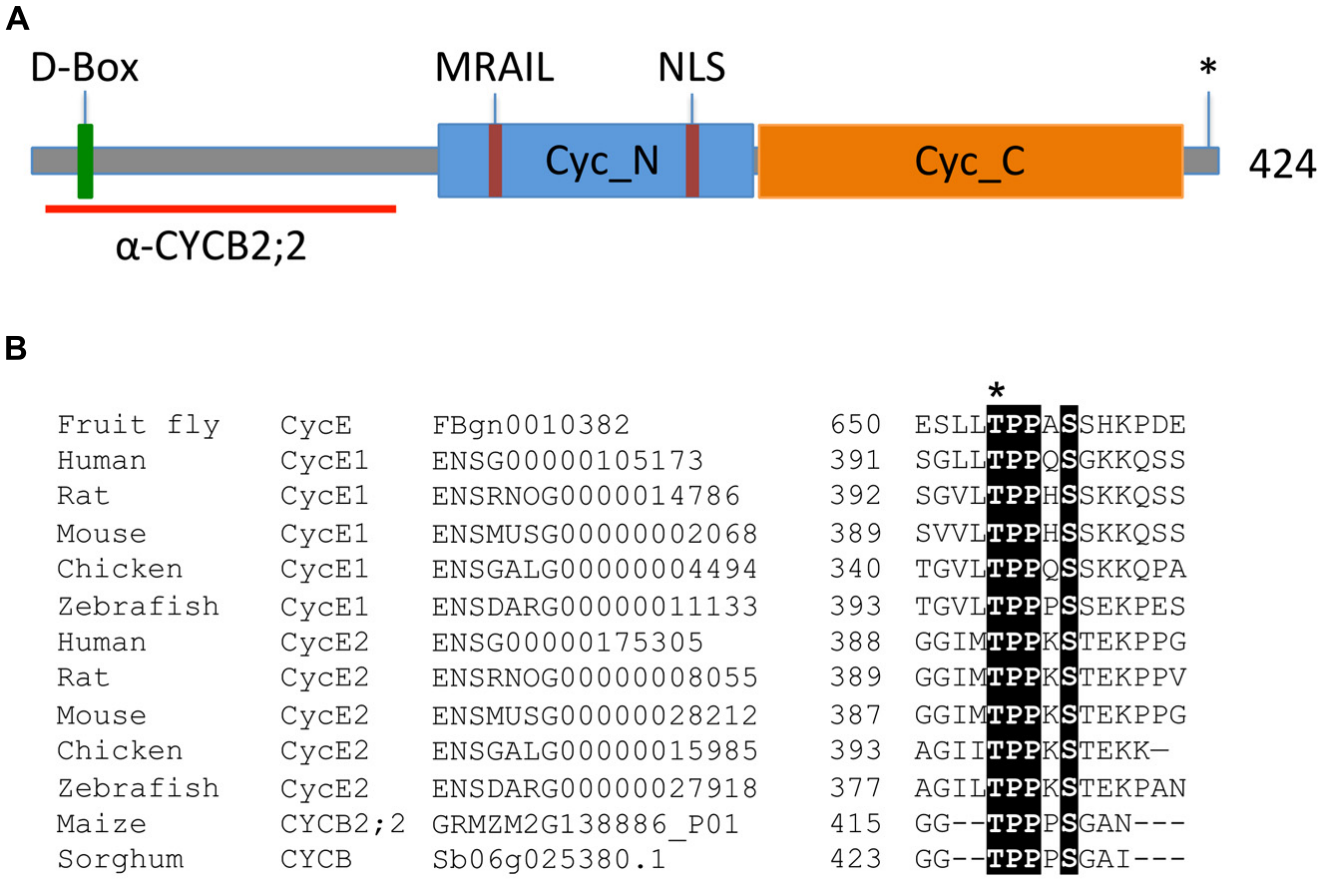
**Key features of the maize CYCB2;2 polypeptide. (A)** Schematic diagram illustrating the conserved Cyc_N and Cyc_C domains, the positions of the destruction box (D-Box), the NLS, the MRAIL motif, and the putative CDK phosphorylation site at position 417 (asterisk), as well as the *N*-terminal region selected for raising specific antibodies (red line). **(B)** Multiple sequence alignment showing the Thr-417 putative phosphorylation site in maize CYCB2;2, which is conserved among animal E-type cyclins and in a closely related protein from sorghum (Sb06g025380.1). The Thr-395 residue in the human CycE1 sequence is often reported as Thr-380 in the literature due to a 15-amino acid shorter isoform of the protein. Conserved residues are shaded. Ensembl database (http://uswest.ensembl.org/) and plant gene IDs are given.

An EST encoding a B-type cyclin from maize, hereafter termed CYCB2;2, was identified by searching Pioneer Hi-Bred Inc.’s database. Subsequently, a corresponding genomic sequence located on chromosome 2, with accession number GRMZM2G138886, was also identified. The deduced CYCB2;2 protein sequence is 424 amino acids long, with a calculated molecular weight of 47.5 kDa. It possesses two all-alpha fold domains embedded in the so-called and highly conserved “cyclin core,” which are characteristic of cyclin proteins: Cyc_N (or cyclin box), which contains the CDK-binding site and is located between residues 163 and 289, and Cyc_C, which however, is less conserved among cyclins, and is located between residues 291 and 407 (Nugent et al., 1991; **Figure 1A**). A potential PRKK nuclear localization signal (NLS) is located between residues 231–234. A protein destruction box (SRRALTDIK, D-Box), targeted by the anaphase-promoting complex/cylosome (APC/C) and which is required for anaphase-specific proteolysis, is predicted at residues 26–34. A MRAIL motif, which is involved in cyclin binding to substrates containing the RxL motif, such as CDK-specific inhibitors and RBRs (Schulman et al., 1998), is located between residues 192–196.

**FIGURE 2:**
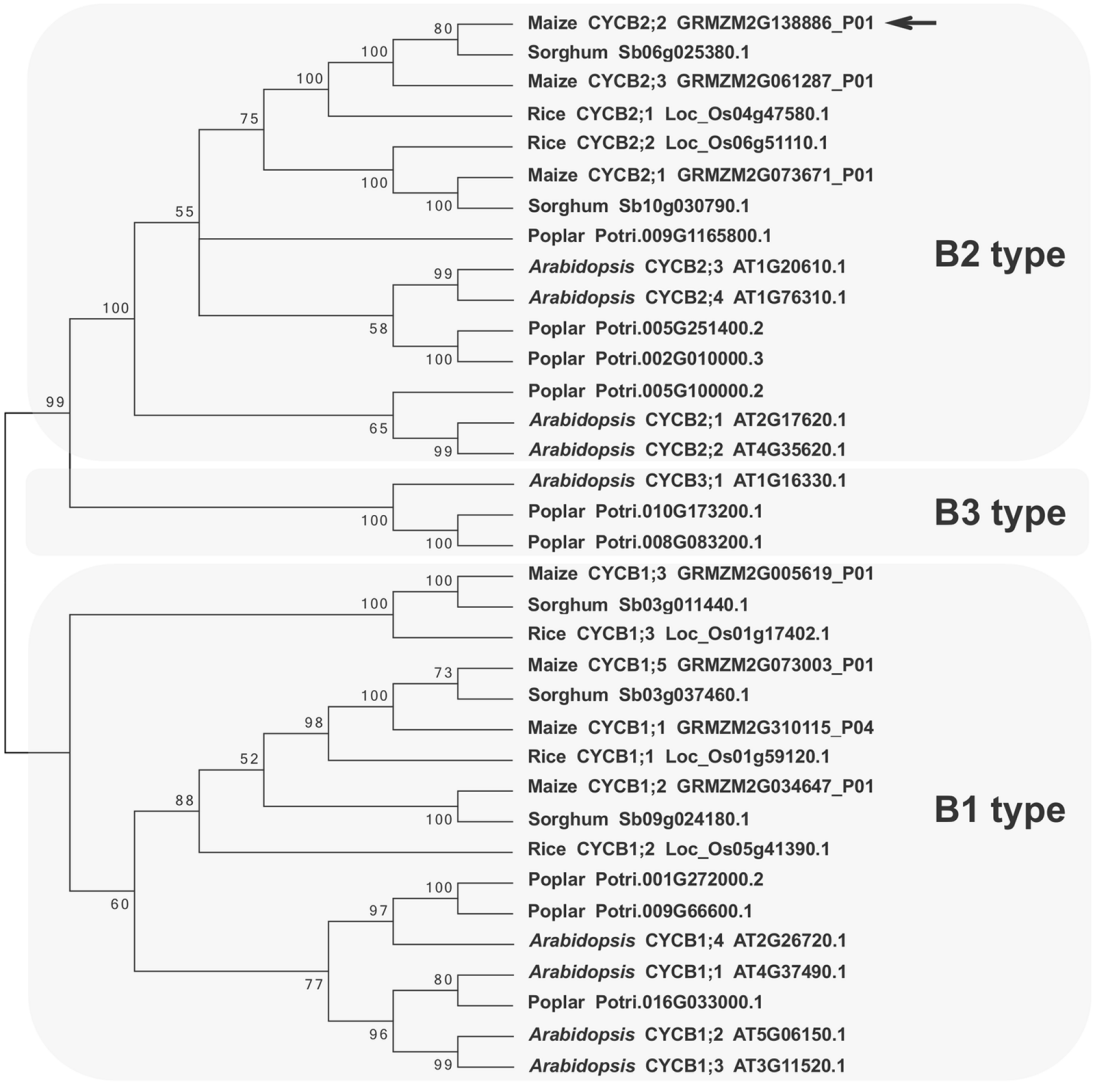
**Phylogenetic tree of B-type plant cyclins.** Selected plant cyclin B amino acid sequences from *Arabidopsis*, poplar, maize, sorghum, and rice were aligned and an un-rooted tree was generated with the Neighbor-Joining method. Bootstraps values (>50) are shown next to the branches. Branches corresponding to partitions reproduced in less than 50% of bootstrap replicates are collapsed. Protein accession numbers and cyclin designations (when available) are given next to the plant species names. Three main cyclin B groups can be identified: B1-, B2-, and B3-type. The maize CYCB2;2 polypeptides described in this study is shown at the top of the B2-type group and is indicated by an arrow. The closely related GRMZM2G061287_P01 polypeptide is a newly identified B2-type cyclin from maize, which was designated as CYCB2;3. Evolutionary analyses were conducted in MEGA6 (Tamura et al., 2013).

Thr-417 represents a potential CDK phosphorylation site, which is conserved with similar phosphorylation sites in animal CycE proteins (**Figures 1A,B**). Phosphorylation of this site contributes to CycE proteolysis via the SCF-Fbw7 ubiquitin ligase pathway (Welker et al., 2003; Hwang and Clurman, 2005; Hao et al., 2007). Through database searches, a closely related cyclin from sorghum (Sb06g025380.1) was identified that also possesses this putative phosphorylation site (**Figures 1B** and **2**), though it does not appear that this motif is conserved in other plant cyclins.

Phylogenetic analyses indicated that the maize CYCB2;2 cyclin described in this study clusters with other B2-type cyclins from monocots (maize, sorghum, and rice) and dicots (*Arabidopsis* and poplar). These results are in general agreement with previous analyses (Hu et al., 2010). During the course of this study, a gene was identified on chromosome 10 (GRMZM2G061287) that encodes a closely related and previously un-reported maize cyclin, which was termed CYCB2;3. The closest cyclins from rice (CYCB2;1) and sorghum (Sb06g025380.1; **Figure 2**) also lack functional characterization to date, which makes it difficult to predict a precise function for maize CYCB2;2 based on sequence similarity.

**FIGURE 3:**
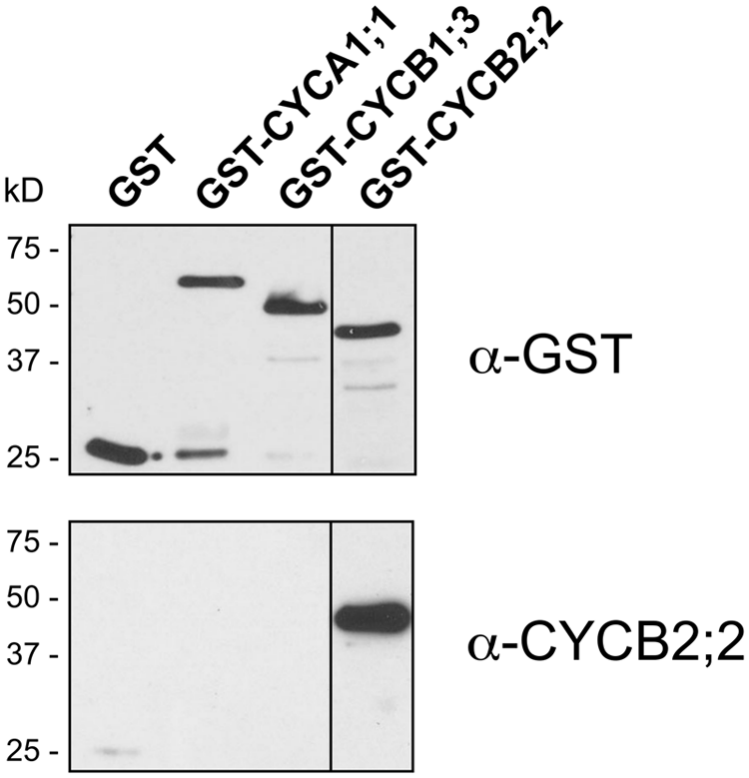
**Specificity of anti-CYCB2;2 antibodies.** Affinity-purified antibodies against CYCB2;2 were tested against the CYCB2;2 antigen and selected domains of maize cyclins CYCA1;1 and CYCB1;3, expressed in *E. coli* as GST fusions. **Top**, control western blot with anti-GST antibodies; **Bottom**, western blot with anti-CYCB2;2 antibodies.

An *N*-terminal region of CYCB2;2 (amino acid residues 4–143) that has little or no sequence identity with other maize cyclins identified so far (∼39% identity with the corresponding regions in CYCB2;1, the closest maize homolog) was selected to raise specific antibodies (**Figure 1A**). This region of the protein lies outside the Cyc_N domain, and thus these antibodies were not likely to interfere with the catalytic or CDK-binding activity of CYCB2;2. The corresponding *CYCB2;2* cDNA sequence was expressed in *E. coli* as a GST fusion and polyclonal antibodies were raised in rabbits against the purified protein, and were affinity purified. These antibodies were tested against the recombinant CYCB2;2 antigen and comparable *N*-terminal regions of maize CYCA1;1 and CYCB1;3, which were also expressed as GST-fusions in *E. coli* as described previously (Dante et al., 2014; **Figure 3**). While these antibodies effectively recognized the CYCB2;2 *N*-terminal polypeptide, no cross-reactivity was observed with either CYCA1;1 or CYCB1;3.

### EXPRESSION OF CYCB2;2 DURING ENDOSPERM DEVELOPMENT

**FIGURE 4:**
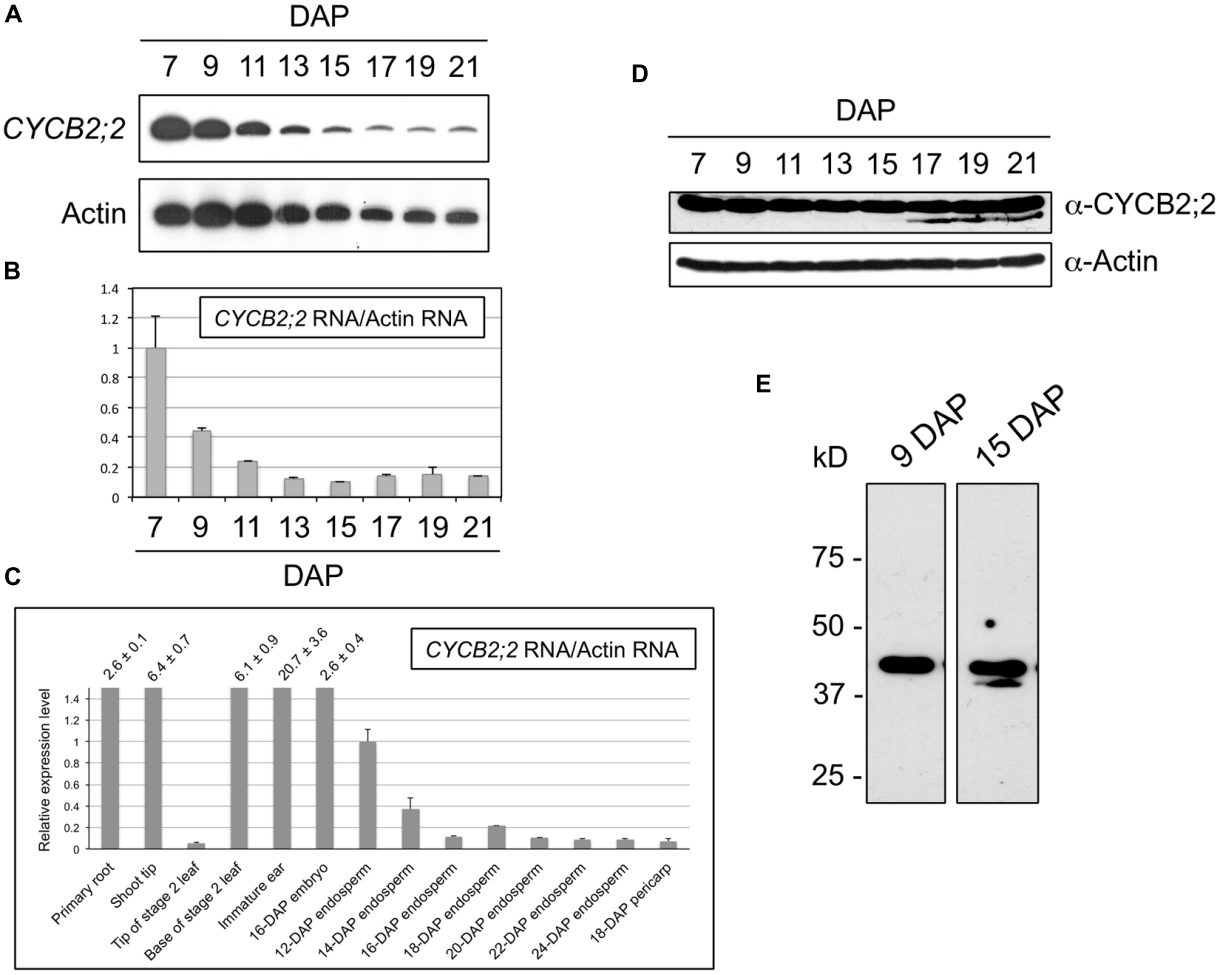
**CYCB2;2 expression patterns during maize endosperm development. (A–C)** Analyses of RNA accumulations patterns. **(A,B)** Analysis of *CYCB2;2* RNA accumulation pattern during endosperm development from 7 to 21 DAP by RT-PCR. Actin RNA was used as a control. Error bars indicate standard deviations. **(C)** Analysis of CYCB2;2 RNA accumulation, relative to that of actin, in 14 maize tissues and developmental stages. Error bars indicate standard deviations. This analysis was based on data from a previous study by Sekhon et al. (2011). **(D,E)** Western blots. Actin expression pattern was used as a control. Accumulation of CYCB2;2 protein is roughly constant, despite decreasing levels of RNA. A lower molecular weight CYCB2;2-related polypeptide accumulated specifically in endoreduplicating (e.g., 15-DAP) endosperm cell extracts **(E)**. The actin blot was part of a set of common experiments carried out during a larger study and was published previously (Sabelli et al., 2005).

The expression patterns of CYCB2;2 RNA and protein during endosperm development were analyzed by RT-PCR and western blotting, respectively (**Figure 4**). These experiments showed that *CYCB2;2* RNA levels are relatively high in 7-DAP endosperm and decline steadily thereafter, reaching by 13-DAP a minimum of less than 20% the 7-DAP reference levels, and remaining low up to 21-DAP (**Figures 4A,B**). This result is in agreement with the expression pattern of *CYCB2;2* RNA obtained through global transcriptome analyses of developing maize endosperm (12–24 DAP) and available through the Maize eFP Browser (Sekhon et al., 2011; **Figure 4C**). Additionally, the data shown in **Figure 4C** indicate that *CYCB2;2* RNA is widely expressed in maize tissues, and particularly at high levels in those known to contain an elevated proportion of mitotic cells, such as the primary root, shoot tip, leaf base, immature ear, embryo, and young endosperm.

The anti-CYCB2;2 antibody recognized a polypeptide of the expected molecular weight in endosperm extracts (**Figure 4D**). However, longer autoradiograph exposure times revealed the presence of an additional band of slightly lower molecular weight, which tended to accumulate specifically during the endoreduplication phase of endosperm development and was detectable from around 13-15 DAP (**Figure 4E**). This low-molecular-weight (LMW) CYCB2;2-related polypeptide was never detected in 7- or 9-DAP extracts; its accumulation appeared to be associated with the endoreduplication phase of endosperm development, which suggests it may play a specific role in this specialized type of cell cycle.

Comparison of the CYCB2;2 RNA and protein accumulation patterns revealed a discrepancy between the marked decline of RNA levels and the relatively constant protein levels observed between 7 and 21 DAP. This observation raises the possibility that CYCB2;2 protein may be subject to different turnover regulation in early (i.e., 7-DAP) *versus* more advanced (i.e., 13–21 DAP) endosperm developmental stages, which primarily comprise cells that are mitotic or endoreduplicating, respectively. Specifically, CYCB2;2 may not be as efficiently degraded in endoreduplicating endosperm as in mitotic endosperm.

### SUBCELLULAR LOCALIZATION OF CYCB2;2 PROTEIN

**FIGURE 5:**
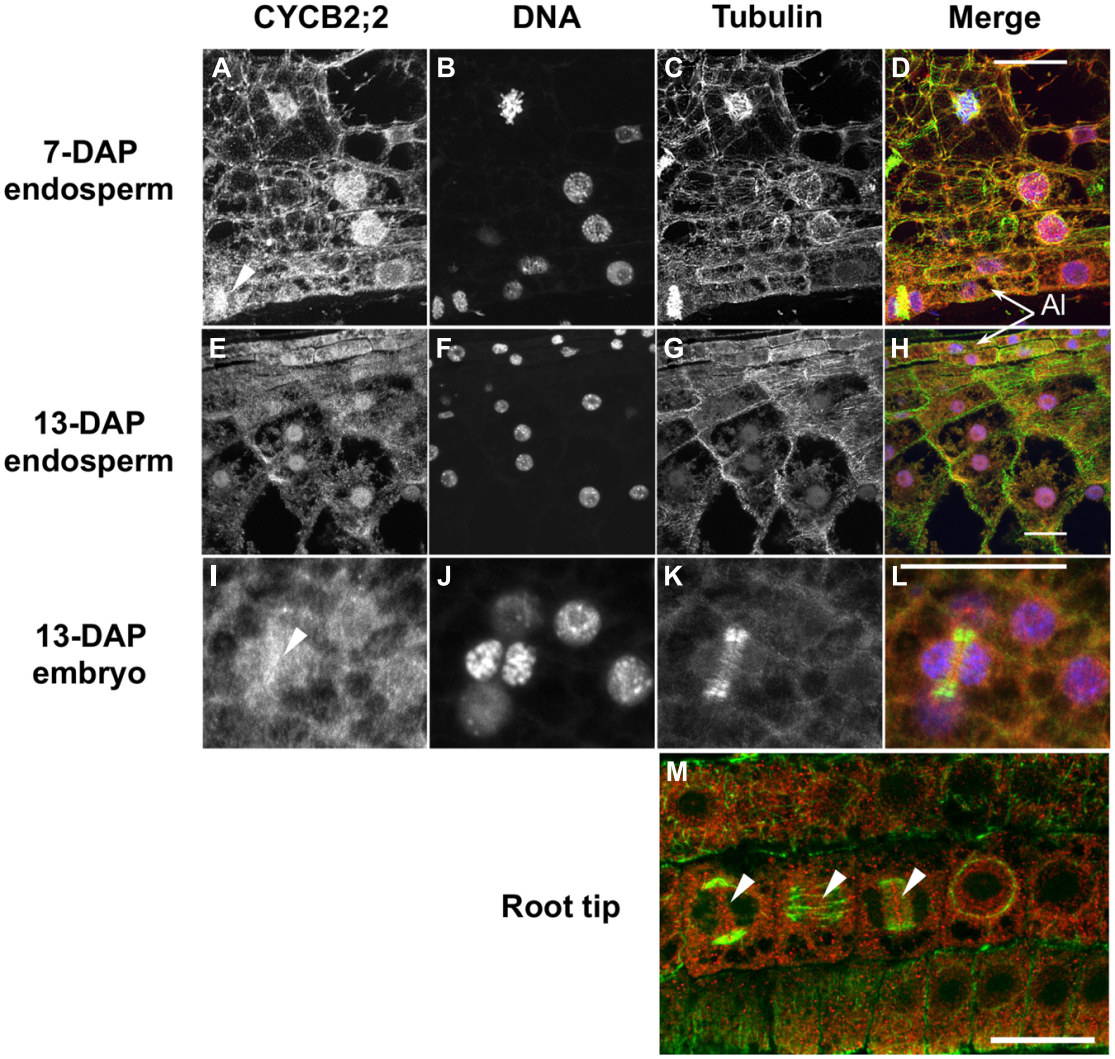
**Immunolocalization of CYCB2;2 protein in maize endosperm, embryo and root tip cells.** Treatments as follows: **(A,E,I)**, anti-CYCB2;2 antibody; **(B,F,J)** DNA staining; **(C,G,K)** anti-tubulin antibody; **(D,H,L)** merge (CYCB2;2, red; DNA, blue; tubulin, green). **(A–D)** 7-DAP endosperm; **(E–H)** 13-DAP endosperm; **(I–L)** 13-DAP embryo. **(M)** Merge image of CYCB2;2 (red) and tubulin (green) staining in root tip cells. Panels **(I–L)** show CYCB2;2 localization (arrow in **I**) in a late-phragmoplast cell. Arrows in **(A**,**I,M)** indicate CYCB2;2 localization to the phragmoplast or cell division site. Arrows in **(M)** indicate CYCB2;2 localization in cells during cytokinesis (left arrow), anaphase (center arrow), and telophase (right arrow). The aleurone layer (Al) is indicated in **(D,H)**. Scale bars = 25 μm.

Sequence analyses by subcellular localization prediction software and the presence of a putative NLS in the CYCB2;2 amino acid sequence suggested this protein could be targeted to the nucleus. We investigated the localization pattern of CYCB2;2 in endosperm, embryo, and root tip cells by immunofluorescence using anti-CYCB2;2 antibody (**Figure 5**). DNA and tubulin were stained as markers for nuclei and the microtubule cytoskeleton, respectively, in the same tissue sections. In 7-DAP endosperm, CYCB2;2 was clearly localized to the nucleus, though a weak signal was also detected in the cytoplasm (**Figures 5A–D**). Although most endosperm nuclei stained positively for CYCB2;2, the signal among nuclei differed notably, with a rather diffused CYCB2;2 accumulation pattern in some nuclei and a sharply punctuate one in others. These differences in localization patterns within a population of cells that are asynchronously engaged in the cell cycle are typical of cell cycle-regulated proteins. In 13-DAP endosperm cells, CYCB2;2 was clearly localized to the nucleus but it also showed extensive and diffuse localization in the cytoplasm, which was clearly more pronounced than in 7-DAP endosperm cells (**Figures 5E–H**). However, peripheral cells gave a relatively stronger CYCB2;2 signal than inner endosperm cells in both 7- (**Figures 5A,D**) and 13-DAP (**Figures 5E,H**) sections, but it is not clear whether this might be due to physiological differences between the two cell types or to the highly dense cytoplasm of peripheral cells at both developmental stages. Analysis of 7-DAP endosperm (**Figure 5A**, arrow indicates a telophase cell), embryo (**Figures 5I–L**, arrow indicates a late-phragmoplast cell in which the residual phragmoplast is being eroded beginning from its central zone) and meristematic root tip cells (**Figure 5M**), which typically proliferate through the mitotic cell cycle and do not undergo endoreduplication, revealed CYCB2;2 was localized to the phragmoplast between two daughter nuclei, suggesting a potential role for CYCB2;2 in late mitosis-early cytokinesis and the deposition of the new cell wall separating daughter cells. In root tip cells (**Figure 5M**), CYCB2;2 was localized to the cytoplasm but also to the mitotic spindle’s mid-zone in anaphase (center arrow), the phragmoplast mid-zone in telophase (right arrow) and at the site of cell wall formation at cytokinesis (left arrow). Both in mitotic and endoreduplicating cells, the nuclear fraction of CYCB2;2 did not appear to coincide with the DNA, and thus it appeared to be generally excluded from chromatin, similarly to CYCB2;1 (Mews et al., 1997), but there was some overlap with the microtubule cytoskeleton.

**FIGURE 6:**
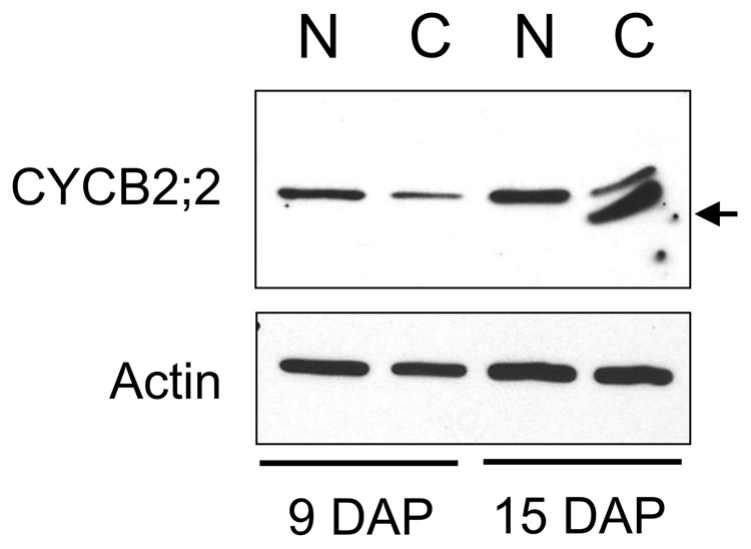
**A low-molecular-weight form of CYCB2;2 accumulates in the cytosol of endoreduplicating endosperm cells.** Extracts were prepared from nuclei- (N) and cytosolic- (C) enriched fractions from 9- and 15-DAP endosperms. Western analysis with anti-CYCB2;2 antibody revealed this protein was prevalently nuclear at 9 and 15 DAP, but a lower molecular weight polypeptide accumulated in the cytosolic fraction at 15-DAP. Anti-actin antibody was used as a control.

We compared the subcellular distribution patterns of CYCB2;2 between 9-DAP endosperm cells, which are mostly mitotic, and 15-DAP cells, which are primarily endoreduplicating. Subcellular fractions enriched for nuclear and cytosolic proteins were prepared from these two endosperm developmental stages and assayed for CYCB2;2 accumulation by western blotting (**Figure 6**). CYCB2;2 appeared prevalently localized to the nuclear fraction in 9-DAP endosperm, but a lower molecular weight polypeptide, corresponding to the additional LMW band shown in **Figures 4D,E**, accumulated specifically in the cytosolic fraction in 15-DAP endosperm. The presence of intact actin protein as well as tubulin and other cyclins as recently reported (Dante et al., 2014) indicates that the LMW, CYCB2;2-related band was not due to general protein degradation in the extract. These results are in agreement with the immunohistochemistry data shown in **Figure 5**, which does not discriminate signal based on molecular weight differences. In prevalently mitotic endosperm (i.e., 7–9 DAP), CYCB2;2 is mostly nuclear with relatively little signal from the cytoplasm. In endoreduplicating cells (i.e., 13–15 DAP), however, CYCB2;2 becomes more extensively localized to the cytoplasm, and the cell fractionation analysis suggests that this shift in localization is due to the specific accumulation of an extra-nuclear, LMW form of the protein (**Figure 6**). The presence of similar amounts of full-length CYCB2;2 in mitotic and endoreduplicating endosperm cells suggests this protein maybe involved in regulating cell cycle processes that are common to these two cell types, such as DNA synthesis. Alternatively, partial CYCB2;2 proteolysis and exclusion from the nucleus may be associated with the endocycle and may underscore a role for the intact protein in mitosis and/or cytokinesis. Thus, in contrast to mitotic cells, endoreduplicating endosperm cells are characterized by specific nuclear-to-cytosol redistributions of CYCB2;2, specifically suggesting that accumulation of the LMW component in the cytoplasm may be critical for the transition from the mitotic to endoreduplication phase of endosperm development.

### CYCB2;2-ASSOCIATED KINASE ACTIVITY

**FIGURE 7:**
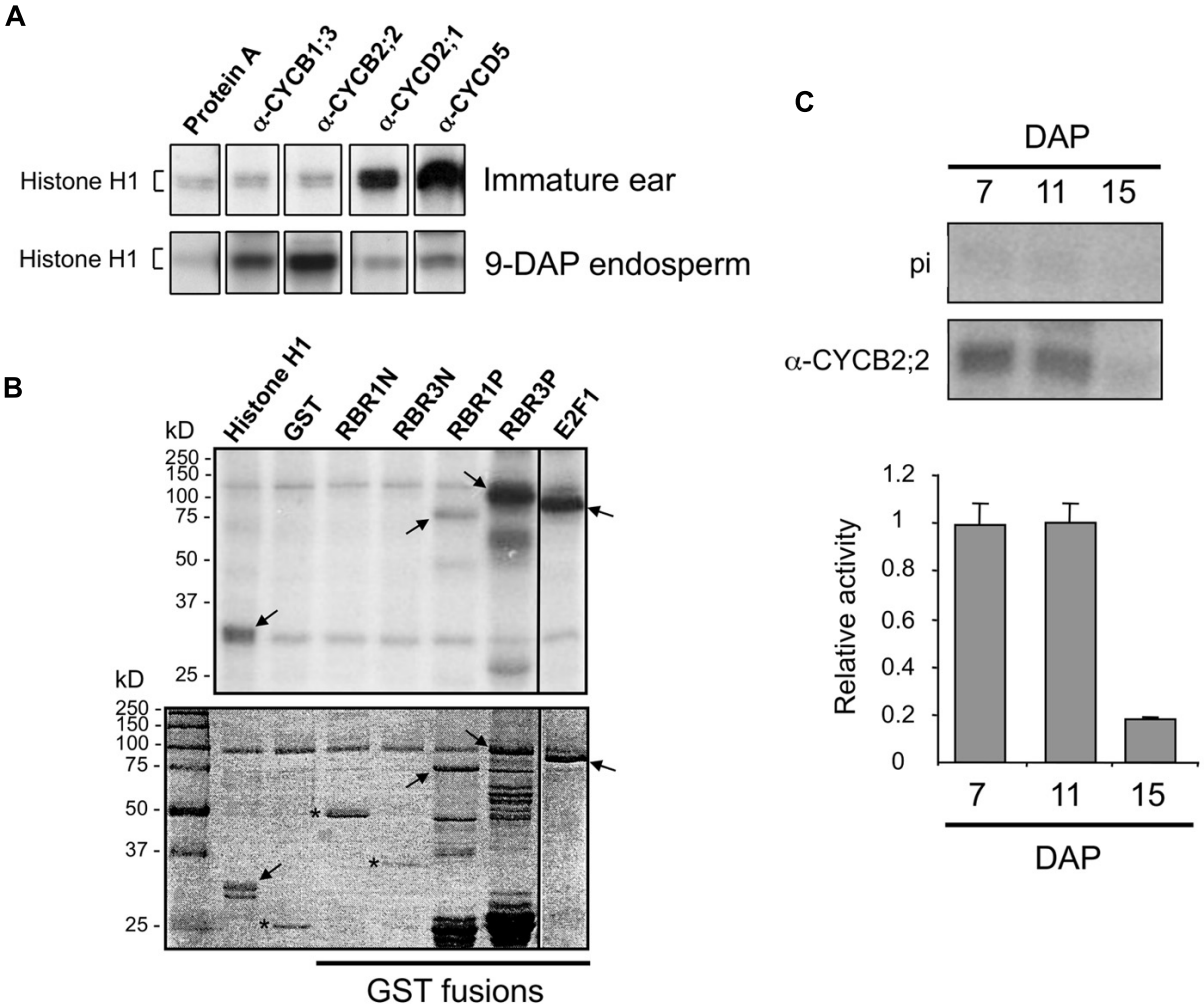
**Analysis of CYCB2;2-associated kinase activity. (A)** Phosphorylation of histone H1 substrate by kinase activities immunoprecipitated from immature ear and 9-DAP endosperm with antibodies against CYCB2;2, and various other cyclins as indicated. Protein A indicates a control reaction carried out without antibody showing background signal due to agarose-Protein A beads. **(B)** Phosphorylation of various recombinant substrates by CYCB2;2-associated kinase activity immunoprecipitated from 9-DAP endosperm (top). Substrates include histone H1, GST, as well as GST fusions of the *N*-terminal domains of RBR1 (RBR1N) and RBR3 (RBR3N), the pocket domains of RBR1 (RBR1P) and RBR3 (RBR3P), and full-length E2F1 (shown in the stained gel, bottom). Arrows indicate specific phosphorylation of substrates. Asterisks indicate substrates that were not phosphorylated (bottom panel). **(C)** Phosphorylation of histone H1 by CYCB2;2-associated kinase immunoprecipitated from 7-, 11-, and 15-DAP endosperm. Top: Authoradiographic detection. Control assays with IgG from pre-immune serum are shown (pi). Bottom: quantitation of kinase activities. Values are averages of two independent experiments, from which background phosphorylation with pre-immune IgG was subtracted. Relative values were normalized by the highest values in each experiment. Error bars represent standard deviations.

We investigated whether CYCB2;2 is part of active kinase complexes, is capable of phosphorylating various substrates, and its activity varies between immature ear and endosperm at different stages of development. CYCB2;2 was immunoprecipitated from extracts prepared with either immature ear and 9-DAP endosperm, two tissues comprising prevalently mitotic cells, and tested for phosphorylation of histone H1 substrate by *in vitro* assays (**Figure 7A**). Whereas virtually no CYCB2;2-associated kinase activity was detected in extracts from immature ear, a strong signal was obtained from 9-DAP endosperm extracts. These patterns were similar to those of CYCB1;3, and antithetic to those of CYCD2;1 or CYCD5, which displayed much strong associated kinase activities in immature ear relative to endosperm. These experiments indicate there are specific differences with regard to the kinase activity associated with CYCB2;2 between immature ears and 9-DAP endosperm, as well as those associated with other types of cyclins. Although both tissues are known to exhibit high mitotic activity, these data underscore the presence of potentially important differences in the regulation of the cell cycle between these two tissues, and suggest a more prominent role in endosperm for B-type cyclins. We next asked whether CYCB2;2-associated kinase activity from 9-DAP endosperm could phosphorylate different recombinant polypeptides expressed as GST fusions (**Figure 7B**). The pocket domain of RBR3 was effectively phosphorylated, although RBR1 pocket was only weakly phosphorylated in this assay. Neither *N*-terminal domain of these proteins was phosphorylated, consistent with the typical predominance of conserved CDK targets specifically within the pocket domain of RBR proteins from many organisms (Sabelli et al., 2005; Sabelli and Larkins, 2009a; Burke et al., 2012). The E2F1 substrate was also phosphorylated by CYCB2;2-associated kinase activity, similarly to recent results for CYCB1;3-associated kinase activity (Dante et al., 2014). By 9-DAP, some endosperm cells could have already initiated the transition to endoreduplication, particularly those located in the center of the tissue, and thus analyses of kinase activity at this stage may not exclusively reflect mitotic cells. We thus measured CYCB2;2-associated kinase activity in 7-DAP endosperm extracts, a stage characterized exclusively by mitotic cells as shown by flow-cytometric profiles (Dante et al., 2014), in 15-DAP extracts, a stage in which cells are actively endoreduplicating and mitotic figures are rare, as well as at 11-DAP, which represents a transition stage with high rates of mitotic and endoreduplication cell cycle activities (Dante et al., 2014). As shown in **Figure 7C**, while CYCB2;2-associated kinase activity was high and virtually at the same levels in 7- and 11-DAP endosperm, it declined dramatically in 15-DAP endosperm. Collectively, these results indicate that CYCB2;2/CDK complexes are more active in mitotic than in endoreduplicating endosperm cells.

**FIGURE 8:**
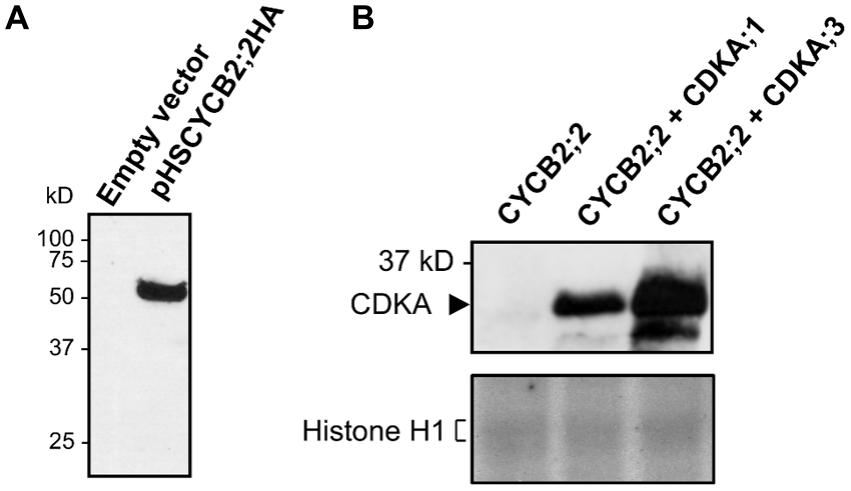
**Interaction of CYCB2;2 with maize CDKs in *Drosophila* S2 cells and analysis of associated kinase activities. (A)** *Drosophila* S2 cells were transfected either with empty pHSKSMCS vector or pHSCYB2;2HA construct, and CYCB2;2HA expression was induced by a 37 °C heat-shock. CYCB2;2HA expression was assessed by western blotting with anti-CYCB2;2 antibody. **(B)** CYCB2;2;HA (CYCB2;2) was expressed in S2 cells either alone or co-expressed with maize CDKA;1 or CDKA;3, and immunoprecipitated with anti-CYCB2;2 antibody. **Top**, Western blot analysis performed on immunoprecipitates with anti-PSTAIR antibody to reveal CYCB2;2-CDKA interaction. **Bottom**, histone H1 kinase activity of immunoprecipitates. Complexes between CYCB2;2 and A-type CDKs show no activity.

We next investigated whether CYCB2;2 can form complexes with A-type CDKs when co-expressed in a heterologous cell system, and whether such complexes are active (**Figure 8**). CYCB2;2 was co-expressed with either maize CDKA;1 or CDKA;3 in *Drosophila* S2 cells. Protein extracts were immunoprecipitated with anti-CYCB2;2 antibody and tested for the presence of either CDKA using anti-PSTAIR antibodies. In both cases, a clear interaction between CYCB2;2 and CDKA;1 or CDKA;3 was detected. However, these CDKA/CYCB2;2 complexes did not phosphorylate histone H1 substrate (**Figure 8B**), suggesting either that the CYCB2;2-associated kinase activity in endosperm extracts is due to a CDK other than CDKA;1 or CDKA;3 or that the expression system utilized for this assay was deficient for some other necessary factors, such as, for example, specific CDK-activating kinases typically acting upstream of CDKAs (Umeda et al., 2000; Shimotohno et al., 2004).

### PROTEASOME-DEPENDENT DEGRADATION OF CYCB2;2 IN ENDOSPERM

**FIGURE 9:**
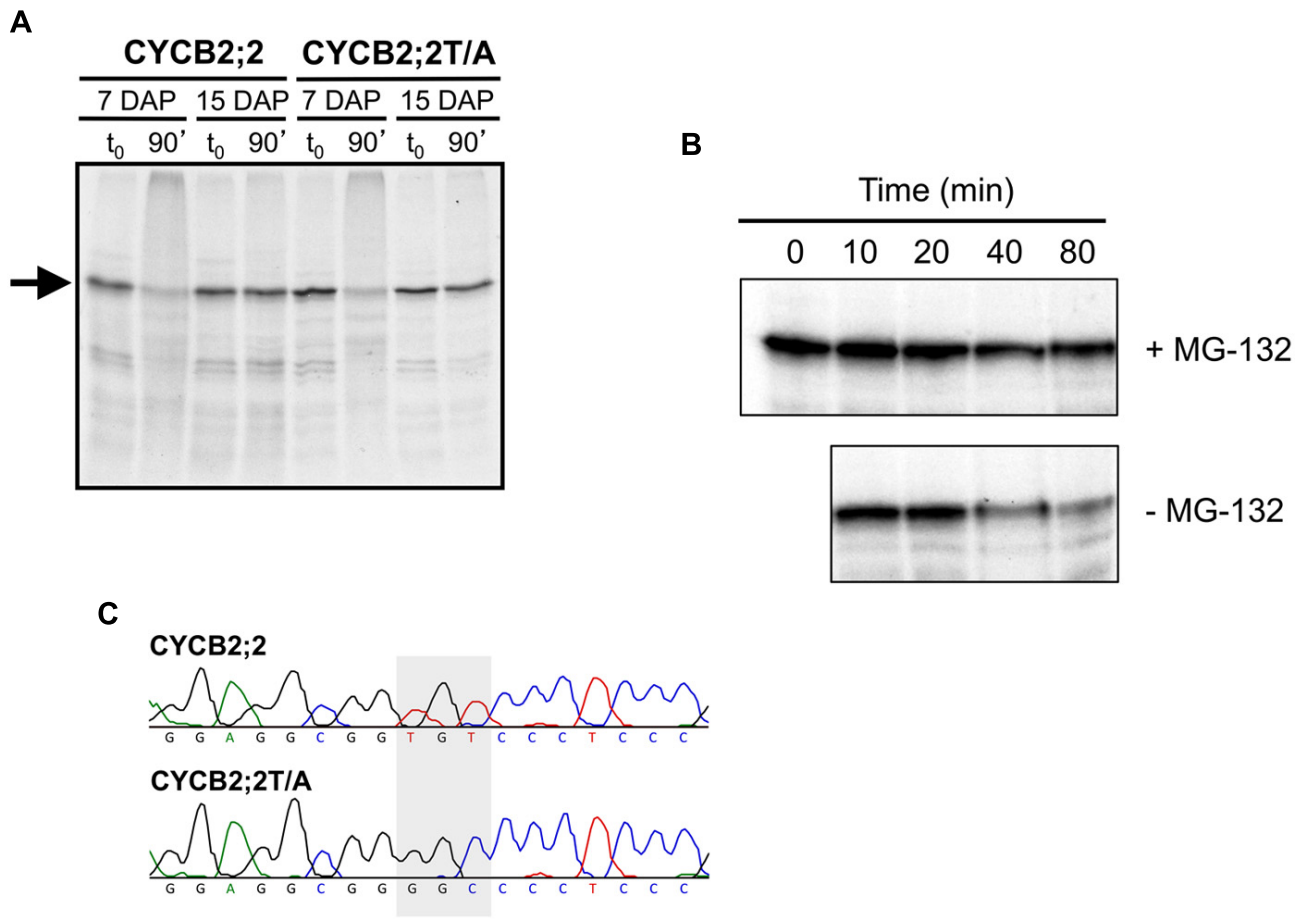
**Analysis of CYCB2;2 stability in mitotic and endoreduplicating endosperm cell extracts. (A)** ^35^S-labeled, *in vitro* synthesized CYCB2;2 and T417A mutant (CYCB2;2T/A) proteins (indicated by an arrow) were incubated for 90 min with mitotic (7-DAP) and endoreduplicating (15-DAP) endosperm extracts, separated by SDS-PAGE and detected by autoradiography. Control reactions, with added endosperm extracts but without incubation, are indicated as t_0_. CYCB2;2 appears to undergo degradation specifically in 7-DAP endosperm extracts, regardless of the T417A mutation. **(B)** Time-course analysis of CYCB2;2 degradation in 7-DAP endosperm extracts in the presence (+) or absence (–) of the proteasome-specific inhibitor MG-132. **(C)** Chromatograms showing the TGT to GGC conversion (indicated by a shaded box) responsible for mutagenizing the potential Thr-417 phosphorylation site in CYCB2;2 protein to Ala-417 in the CYCB2;2T/A protein.

The sustained accumulation of CYCB2;2 protein during endosperm development, in spite of rapidly declining levels of its RNA, suggested that perhaps CYCB2;2 was becoming resistant to degradation by the 26S proteasome during the endoreduplication phase. Indeed, we recently showed that endoreduplicating endosperm can be differentiated from mitotic endosperm by virtue of sustained expression of certain A-, B-, and D-type cyclins associated with their reduced ubiquitin-mediated proteolysis (Dante et al., 2014). A similar outcome was obtained for CYCB2;2 in the present investigation (**Figure 9**). *In vitro*-synthesized CYCB2;2 was almost entirely degraded by a 90-min incubation with mitotic (i.e., 7-DAP) endosperm extracts, whereas it was unaffected by endoreduplicating (i.e., 15-DAP) extracts (**Figure 9A**). Degradation by mitotic extracts at 7-DAP was primarily due to the 26S proteasome, as it could largely be prevented by the addition of the proteasome-specific inhibitor, MG-132 (**Figure 9B**). In addition, we tested whether Thr-417 is required for CYCB2;2 proteolysis. This residue, in fact, is a potential CDK phosphorylation site that is conserved in animal CycE proteins and is necessary for protein degradation via the ubiquitin-dependent pathway (Welker et al., 2003; Hwang and Clurman, 2005; Hao et al., 2007). Thr-417 was mutagenized to Ala-417 (**Figure 9C**), which would destroy the putative phosphorylation site in CYCB2;2, but the mutant CYCB2;2T/A protein was degraded by incubation with mitotic extracts just as effectively as the wild-type protein (**Figure 9A**), suggesting that Thr-417, at least *in vitro*, is not required for CYCB2;2 proteolysis.

## DISCUSSION

Cyclin proteins of the A-, B, and D-type are essential components of CDK complexes that drive the plant cell cycle. Specifically, they contribute to the regulation of the rhythmic activation of CDK activity during the cell cycle and help determine substrate specificity. Here we report the characterization of a B2;2-type cyclin from maize during the development of the seed endosperm, which, similarly to other cereals, typically comprises two successive phases characterized by cell proliferation through the mitotic cell cycle and endoreduplication, which is associated with cell expansion and the growth of the whole tissue.

The temporal expression pattern of CYCB2;2 is characterized by relatively high RNA levels during the mitotic phase of endosperm development (i.e., 7–9 DAP) and a dramatic decline during the endoreduplication phase (i.e., 13–21 DAP). By 13-DAP, the *CYCB2;2* RNA level is less than 20% that of the 7-DAP level, and remains below this figure throughout the period of endosperm development studied up to 21-DAP. This pattern is similar to those of CYCA1;1, CYCA1;2, and CYCB1;3 and clearly distinct from those of D-type cyclins, which display less marked differences in RNA levels between mitotic and endoreduplicating endosperm (Dante et al., 2014). This observation suggests that the primary endosperm role of CYCB2;2 may be in the mitotic cell cycle, which is also supported by the high levels of its RNA observed specifically in mitotic tissues in the Sekhon et al. (2011) dataset. However, when the pattern of accumulation of the encoded protein was analyzed using specific antibodies, it was found the protein is present at roughly constant levels in 7- through 21-DAP endosperm. This pattern is reminiscent of that recently described for CYCB1;3 (Dante et al., 2014), and suggests that the activity of these B-type cyclins is sustained in endoreduplicating cells. Interestingly, a CYCB2;2 immunologically related polypeptide of slightly lower molecular weight accumulated specifically in endoreduplicating but not mitotic endosperm cells, suggesting it may have a specific function during endoreduplication. It is not known whether this LMW protein represents an incomplete form of CYCB2;2 due, for example, to alternative RNA splicing (though there is no evidence of such an occurrence in genomics datasets), the specific presence/absence of phosphate groups, or partial proteolysis, although the latter is unlikely since we found no evidence of CYCB2;2 degradation in endoreduplicating endosperm extracts. An alternative possibility is that this LMW polypeptide reflects the accumulation of another immunologically cross-reactive cyclin. The anti-CYCB2;2 antibodies used in this study do not cross-react with CYCB1;3 or CYCA1;1. The two most closely related maize proteins to CYCB2;2 are CYCB2;1 and the CYCB2;3 polypeptide identified in this study. CYCB2;1 has a calculated MW of 50 kDa, which is larger than that of CYCB2;2 and shares only 39% sequence identity with CYCB2;2 over the *N*-terminal region selected to raise antibodies. The *N*-terminal region of CYCB2;3, on the other hand, is 78% identical to that of CYCB2;2, which suggests potential antibody cross-reactivity between these two cyclins, but CYCB2;3 is slightly larger than CYCB2;2 (426 amino acid-long with a calculated MW of 47.7 kDa). Thus, the above features of CYCB2;1 and CYCB2;3 make it unlikely for either of them, at least as a full-length protein, to account for the CYCB2;2 LMW polypeptide detected in endoreduplicating extracts. Additionally, data on RNA levels available at the Maize eFP Browser (Sekhon et al., 2011) indicate that *CYCB2;1* and the newly identified GRMZM2G061287_T01 (encoding CYCB2;3) transcripts decrease dramatically in the endosperm after 12-DAP, which does not support the idea they could encode the LMW CYCB2;2 band. Thus, unraveling the identity of this LMW CYCB2;2 polypeptide will require further investigation.

Analysis of sub-cellular fractions revealed CYCB2;2 is primarily a nuclear protein in both mitotic and endoreduplicating endosperm. The LMW polypeptide, however, accumulated specifically in endoreduplicating endosperm extracts, confirming data from whole cell extracts, but was solely detected in the cytosolic fraction. Clearly, whatever the identity of this protein, its accumulation pattern suggests it may play a role in determining whether endosperm cells engage in the mitotic or endoreduplication cycle. However, a possible role for this polypeptide in other cytoplasm-associated aspects of endosperm development linked to endoreduplication, such as cell expansion, storage compound deposition and grain filling, cannot be ruled out.

Based on immunohistochemical analyses, CYCB2;2 appears to be localized primarily to the nucleus of mitotic endosperm cells, whereas by 13-DAP it is also distributed extensively throughout the cytoplasm. These data are in agreement with the results from the subcellular fractionation analysis, and presumably a large proportion of the extra-nuclear signal in cells of endoreduplicating endosperm sections is due to the accumulation of the LMW form of the protein. Considerable overlap between CYCB2;2 localization and the microtubule cytoskeleton was consistently observed, which suggests that this cyclin is involved in regulating aspects of cytoskeleton structure and organization, in agreement with previous observations (Mews et al., 1997; John et al., 2001; Weingartner et al., 2004). In dividing cells, whether in the endosperm, embryo or the root tip, CYCB2;2 appeared to accumulate preferentially at the intervening domains between daughter chromatin and nuclei, and it was closely associated with the phragmoplast and with the sites of deposition of the cell plate and nascent cell walls. CYCB2;2 is clearly localized to the residual phragmoplast as it is gradually eroded at cytokinesis (**Figures 5I–L**), further suggesting a role in cell wall formation and cell division. These patterns resemble those of KNOLLE (a cytokinesis-specific syntaxin required for cell plate formation) and NACK1 (a kinesin-like protein involved in the vesicular traffic required for phragmoplast outgrowth; Weingartner et al., 2004), although a large number of proteins are also known to be associated with the phragmoplast and/or the cell plate (McMichael and Bednarek, 2013).

These observations suggest a role for CYCB2;2 in cytokinesis and cell wall formation. Previous data on the expression patterns and accumulation of plant B-type cyclins indicate a mitotic role, but they are contrasting with regard to an involvement in cytokinesis. Typically, the expression profiles of B-type cyclins display a peak in early mitosis, due to transcriptional up-regulation at the G2/M-phase transition coupled to proteolysis during anaphase, and their destruction in late M-phase is essential for proper microtubule cytoskeleton re-organization in late mitosis and cell cycle progression. Over- or ectopic expression of at least some B1- and B2-type cyclins stimulate M-phase entry and cell division (Doerner et al., 1996; Schnittger et al., 2002; Lee et al., 2003; Weingartner, 2003). CYCB1;1 from tobacco and CYCB2;2 from rice associate with metaphase chromosomes and are degraded thereafter (Criqui et al., 2000; Lee et al., 2003; Weingartner, 2003). Tobacco CYCB2;2 is also degraded after prophase (Weingartner, 2003). In maize, CYCB1;1 is prevalently nuclear prior to mitosis, whereas CYCB1;2 is associated with the pre-prophase band, condensed chromosomes and the mitotic spindle and is degraded at anaphase, while CYCB2;1 is predominantly nuclear and becomes associated with the spindle and the phragmoplast at telophase (Mews et al., 1997). The persistence of maize CYCB2;2 (and CYCB2;1 – Mews et al., 1997; John et al., 2001) at the phragmoplast and the site of cell plate formation during telophase clearly contrasts with the typical degradation of most B-type cyclins after metaphase (Bulankova et al., 2013). These differences highlight likely functional specialization among different members within the cyclin B family and its subfamilies (John et al., 2001), as recently confirmed by a large study in *Arabidopsis* (Bulankova et al., 2013). Indeed, we found that maize CYCB2;2 possesses a D-Box and undergoes proteasome-dependent degradation in mitotic endosperm extracts, suggesting it may be destroyed, like other related cyclins, late in mitosis. However, our immunolocalization results indicate that the protein remains present during anaphase and telophase, suggesting that the observed proteasome-dependent degradation is part of the normal turnover regimen for this protein and may not be related to M-phase exit. Interestingly, the apparent ability of maize CYCB2;2 to elude degradation by the proteasome in endoreduplicating endosperm is reminiscent of the outcome of expressing a non-degradable form of tobacco CYCB1;1 (with a non-functional D-Box), which resulted in microtubule disorganization, inhibited phragmoplast formation and caused endomitosis and increased DNA content (Weingartner et al., 2004). It is tempting to postulate that partial proteolysis of CYCB2;2 is responsible for its exclusion from the nucleus in endoreduplicating endosperm cells, which thus would be unable to divide, resulting in an accumulation of the LMW form of the protein in endoreduplicating endosperm. This interpretation is in agreement with the phosphorylation-dependent redistribution of mammalian CycD1 to the cytoplasm (Diehl et al., 1998) and is consistent with the localization of CYCB2;2 to the phragmoplast in mitotic embryo, 7-DAP endosperm, and root tip cells, which is suggestive of a role in cytokinesis. However, redistribution of D1 cyclin to the cytoplasm is coupled with its degradation (Diehl et al., 1998), whereas we did not observe any evidence for proteolysis of CYCB2;2 in endoreduplicating cells. On the contrary, it appears that, like other cyclins, CYCB2;2 is specifically stabilized in endocycling cells (Dante et al., 2014). However, although our analyses of proteolytic activities revealed a clear difference between mitotic and endoreduplicating endosperm in carrying out CYCB2;2 degradation, they are based on a substrate synthesized *in vitro*, which could lack some co- or post-translational modification essential for targeting the protein for proteolysis in a physiological context.

CYCB2;2-associated kinase activity is relatively high in 9-DAP endosperm but only slightly above background levels in immature ear, which is similar to the situation for another maize B-type cyclin, CYCB1;3. In contrast, the kinase activities associated with CYCD2;1 and CYCD5 are high in immature ear and relatively low, though appreciably higher than background, in endosperm. Although both tissues are known to be prevalently mitotic, these differences point to some additional developmental roles specific for D-type cyclins in immature ear and for B-type cyclins in the endosperm. In particular, the kinase activity associated with CYCB2;2 in 9-DAP endosperm can phosphorylate *in vitro* substrates, such as RBR1, RBR3 and E2F1, which are part of the CDK–RBR–E2F pathway controlling the G1/S-phase transition and DNA replication (Sabelli et al., 2013). The presence of a MRAIL motif in the CYCB2;2 sequence is consistent with the observed phosphorylation of RBRs by CYCB2;2/CDK complexes. However, it is not currently known whether these substrates are actual targets of CYCB2;2-associated kinase *in vivo*. Such a kinase activity is sustained at relatively high levels between 7 and 11 DAP, and drops dramatically by 15-DAP, a stage at which starchy endosperm cells are almost exclusively endoreduplicated. The much lower kinase activity at 15-DAP suggests that CYCB2;2/CDK complexes do not significantly participate in the regulation of endoreduplication cycles. In contrast, CYCB1;3-associated activity is very low at 7-DAP, reaches a peak at 11-DAP, and drops only marginally by 15-DAP (Dante et al., 2014). Clearly the kinase activities associated with CYCB1;3 and CYCB2;2 have distinct patterns during endosperm development, suggesting that CYCB1;3, but not CYCB2;2, may also play a role in the endocycle. In insect cells, CYCB2;2 forms complexes, albeit inactive, with CDKA;1 and CDKA;3. CDKA;1 protein has not been found associated with the phragmoplast or the cell plate in maize root tip cells (Colasanti et al., 1993), and thus it is unlikely that it forms active complexes with CYCB2;2 to regulate microtubule dynamics during late mitosis and cytokinesis. Interaction between CYCB2;2 and a B-type CDK (CDKBs specifically regulate the G2/M-phase transition and suppress the endocycle in mitotic *Arabidopsis* cells – Boudolf et al., 2004, 2009), and formation of active kinase were indeed shown in rice (Lee et al., 2003). It remains to be seen whether these interactions occur in a physiological context in maize (i.e., endosperm cells) and whether they result in phosphorylation of downstream targets. However, it is tempting to speculate that inactivation of CYCB2;2/CDK complexes could contribute to down-regulating M-phase-specific kinase activity, thereby licensing endosperm cells for endoreduplication.

Together, these results suggest that CYCB2;2 may possess some specific function particularly associated with mitotic endosperm. However, it is intriguing that this protein and the closely related LMW polypeptide accumulate during the endoreduplication phase of endosperm development, although they apparently possess little, if any, associated kinase activity at this developmental phase. Evidence for a general decrease in proteasome-dependent proteolysis of cyclins that contributes to their stabilization during endosperm development has recently been obtained (Dante et al., 2014). Furthermore, comprehensive analyses have shown there is generally a weak correlation between RNA and proteins levels in maize seeds, and that proteins can disproportionally accumulate in relation to low RNA levels (Walley et al., 2013). The above paradigms seem to hold true also for CYCB2;2, and whether this protein has any role in post-mitotic endosperm development remains to be established.

## Conflict of Interest Statement

A patent application has been filed on the maize CYCB2;2 nucleotide and protein sequences.

## Footnotes

1 http://www.maizesequence.org

2 http://www.maizegdb.org/

3 http://phytozome.jgi.doe.gov

4 http://rice.plantbiology.msu.edu/

5 http://www.gramene.org/

6 http://pfam.xfam.org/

7 http://bar.utoronto.ca/efp_maize/cgi-bin/efpWeb.cgi?dataSource=Sekhon_et_al

8 http://elm.eu.org/

9 https://rostlab.org/services/loctree3/

10 http://www.csbio.sjtu.edu.cn/bioinf/plant-multi/

11 http://gpcr.biocomp.unibo.it/bacello/

12 http://tcoffee.crg.cat/apps/tcoffee/index.html
